# Determinants of an HIV Preventive Vaccine among a Highly Vulnerable Population: African American Men Who Have Sex with Men

**DOI:** 10.3390/vaccines12030323

**Published:** 2024-03-18

**Authors:** Mia Ann Xu, Jasmin Choi, Joshua G. Rosenberger, Rick S. Zimmerman, Ralph DiClemente

**Affiliations:** 1 Department of Social and Behavioral Sciences, School of Global Public Health, New York University, New York, NY 10003, USA; jasmin.choi@nyu.edu (J.C.); rjd438@nyu.edu (R.D.); 2 College of Health and Human Development, Penn State University, State College, PA 16801, USA; jgr17@psu.edu; 3 College of Nursing, Wayne State University, Detroit, MN 48202, USA; rickzimmerman@wayne.edu

**Keywords:** HIV, vaccine hesitancy, MSM, African American

## Abstract

African American men who have sex with men (MSM) are disproportionately impacted by HIV and may benefit from the development of an HIV vaccine. African American MSM are adversely affected by discrimination as a function of both their race and sexual behaviors. This may further increase the challenges associated with persuading them to adopt an HIV vaccine. Developing a knowledge base characterizing African American MSM HIV vaccine perceptions, attitudes, and concerns may help strengthen how healthcare providers and other health stakeholders describe and discuss the advent of an HIV vaccine. This study assessed the knowledge, attitudes, beliefs, and intentions related to HIV vaccination among African American MSM. This study comprised 432 African American MSM, 18–64 years, residing in the United States. Vaccine intention was defined as how likely it is that an individual would adopt an HIV vaccine if a vaccine was available and it was 90% effective against HIV, easy to obtain, free, and had few side effects. Relative to African American MSM who intend to delay receiving an HIV vaccination, controlling for age, education, and income, early vaccine adopters who had received ≥ 2 COVID-19 vaccinations and who had high WHO HIV Vaccine Positive Attitude Scale scores were, respectively, 3.2 times and 2.4 times more likely to report the intention to vaccinate within one year. Early vaccine adopters were also 2.4 times more likely to feel that HIV prevention support discriminates against African American MSM. Those reporting three or more sexual partners and medical mistrust were, respectively, 60% and 59% more likely to report the intention to delay HIV vaccination. The lack of a knowledge base on HIV vaccine perceptions and acceptability is a missed opportunity to provide guidance on how stakeholders, such as health providers and policymakers, should address HIV vaccine hesitancy once this crucial vaccine is licensed. The key factors affecting vaccine adoption are valuable in developing and implementing campaigns to enhance the HIV vaccine coverage in this vulnerable population.

## 1. Introduction

Human immunodeficiency virus (HIV) remains a serious global health threat. As of 2023, 39 million people were living with HIV, with 1.3 million infections occurring annually [1]. Despite virally suppressive antiretroviral treatment, people with HIV face greater chronic non-infectious diseases and have comorbidities sooner and more frequently than the general population, attributable, in part, to treatment side effects and residual inflammation or immune activation [2]. In terms of prevention, PrEP (pre-exposure prophylaxis) has proven effective at reducing the risk of HIV transmission. However, PrEP remains a costly long-term strategy [3,4,5]. Consequently, the optimal HIV prevention strategy may be a preventative HIV vaccine [6].

Despite the dedication of health organizations, governments, and the scientific community, there has yet to be a safe and effective licensed HIV vaccine [7,8]. However, new biomedical advances, such as the COVID-19 vaccine, which was developed rapidly based on an mRNA platform, along with advances in vaccinology, have galvanized the medical community and hold promise for the development of an HIV vaccine [8,9]. Currently, several mRNA-based HIV vaccine candidates are in clinical trials [10]. The development of a safe and effective vaccine may markedly reduce HIV acquisition, which, in turn, would reduce the transmission rate of HIV. However, even with a licensed HIV vaccine, enhancing the uptake among the most vulnerable populations may be challenging.

Some populations are disproportionately impacted by HIV and may show the greatest benefit from an HIV vaccine. In the United States, nearly half of new HIV infections occur among African American men who have sex with men (MSM) [11]. Several factors may adversely affect African American MSM from receiving an HIV vaccine. Discrimination based on their race and sexual orientation may increase their vulnerability to accessing clinical services, including receiving a vaccine [12,13,14]. Given the burden of disease among African American MSM, a vaccine may be critical to reducing HIV transmission among this marginalized and underserved community [15].

An important public health lesson from the global response to the COVID-19 pandemic was that developing a vaccine is only the initial challenge; a persistent challenge is encouraging people to accept the vaccine [16,17]. The WHO’s Strategic Advisory Group of Experts (SAGE) on Immunization recognized that it is essential to address vaccine attitudes before people start doubting or refusing an actual vaccine [18,19,20]. Understanding HIV vaccine intention predictors enables health stakeholders to better understand and address the HIV vaccine concerns and needs to create equitable HIV vaccine provision and reduce disparate HIV outcomes. In the absence of vaccine preparedness studies, there is limited information for characterizing the population’s HIV vaccine perceptions and concerns. Thus, we may be missing an opportunity to help strengthen our public health response should an HIV vaccine become available.

This study examined the knowledge, attitudes, beliefs, and intentions related to HIV vaccination among an online, recruited sample of African American MSM.

## 2. Methods

This observational study used data from an online survey to examine HIV vaccination knowledge, attitudes, and intentions. To ensure that questions were relevant and culturally appropriate, prior to developing the survey, semi-structured interview guides pertaining to the study questions and study protocol were reviewed by a small sample of African American MSM; focus groups were also conducted with African American MSM at AIDS service organizations.

The study was completed among African American MSM during August of 2023.

### 2.1. Data

Survey respondents were recruited from virtual advertisements placed on Facebook. The advertisements on the platforms were targeted towards users based on them currently being in the United States and identifying as male and LGBTQ+. The study team developed a total of 5 advertisements, and the ads ran continuously for a two-week period. The ads were viewed by (i.e., reached) a total of 33,312 individuals. The survey was administered via Qualtrics, a survey and data collection tool. Qualtrics, as opposed to other data collection tools, was recommended by the IRB to be the data collection tool for this study due to its higher level of security.

Upon clicking the advertisements, potential respondents were directed to a Qualtrics study webpage. The survey questionnaire included two parts. Part one of the survey determined a potential respondent’s eligibility. Once the participant was assessed as eligible based on part one of the survey, the participant was invited to an electronic link to complete the second part of the survey. The eligibility criteria for participation in the study included the following: (1) the ability to speak and read English; (2) primary place of residence in the United States; (3) 18 years of age or older; (4) self-reported biological sex with males; (5) self-reported race of African American; (6) an HIV-negative serostatus; (7) sex with at least two other males within the past year; and (8) reported non-condom use in the past year.

If a respondent satisfied the eligibility criteria, they responded to part two of the survey. Part two included 145 structured items. The survey assessed respondents’ sociodemographic statuses (e.g., age, education, income), sexual behaviors, health behaviors, key theoretical constructs relevant to vaccine adoption, perceptions pertaining to sexual orientation and discrimination, perceptions about HIV and HIV vaccines, COVID-19 vaccine histories, and medical mistrust. Participants who met the study eligibility criteria and completed the survey were compensated with a USD 20 Amazon gift card sent to the email address they provided in part one of the survey; this part was not linked to the second part of the survey that collected data to address the study aims. New York University’s Institutional Review Board approved all study protocols (IRB-FY2022-6665).

### 2.2. Recruitment

The research team filtered 2165 survey respondents based on the following: duplicate I.P. addresses (*n* = 772); those not meeting all of the study eligibility criteria (*n* = 229); those whose geographic locations were not consistent with the state in the U.S. that they reported residing in on the survey (*n* = 560); those who completed the survey in less than half of the median completion time (*n* = 91); those who reported that they “were less than very honest” in their survey responses (*n* = 10); and those who were able to access and complete part two of the survey without completing part one of the survey first (*n* = 71). The final survey sample included 432 respondents based on the filtering methods above.

### 2.3. Survey Measures

Respondents completed theoretically and empirically relevant measures in the following categories: demographics, health behavior, sexual behavior, theoretical constructs associated with vaccine adoption, perceptions about sexual orientation and discrimination, history of COVID-19 vaccine uptake, HIV perceptions, and perceptions especially related to HIV vaccines.

Through a theory-driven approach, several psychosocial models that have been understood to enhance behavior change for vaccine intentions served as the theoretical underpinnings for the survey. One model was the Health Belief Model (HBM). The HBM focuses on an individual’s beliefs regarding the presence of a threat and the potential actions taken to address that threat [21,22]. The barriers and individual perceptions that influence action are critical components of the HBM, which is used to predict decisions, such as the intention to vaccinate among African American MSM [23]. The HBM was supplemented with several items adapted from the Extended Parallel Processing Model [24].

#### 2.3.1. Outcome Variable: Vaccine Intention

Vaccine intentions were derived from previous vaccine research [25]. Vaccine intention included the following: “If a new HIV vaccine was available and it was 90% effective against HIV, is easy to get, free, and has few side effects, how likely would you do each of the following”. Respondents completed six intention questions: “Get the vaccine”; “Get the vaccine immediately”; “Get the vaccine in the first 3 months”; “Get the vaccine in the first year”; “Get the vaccine after more than a year”; and “Wait until I see how the vaccine is going with other people before deciding if I should get the vaccine”. Each of the six intention questions was based on 4 categories: (1) I will definitely do this; (2) I will probably do this; (3) I will probably NOT do this; and (4) I will definitely NOT do this. Summing across the six questions, we created a composite summary score that reflects each respondent’s intention to vaccinate.

Factor analysis was used to determine the early-intention variables. We observed a two-factor loading. Within factor 1, we did a median split between immediate adopters compared to those who would wait one year or more. We identified which items looked at early vaccine intentions and determined that factor loadings greater than or equal to 0.7 were included as the early-vaccine-intention group [26]. The early-vaccine-intention variables included “Get the vaccine immediately”, “Get the vaccine in the first 3 months”, and “Get the vaccine in the first year”, and they were averaged to create a single variable: the “Early intention to vaccinate”. Within the early intention to vaccinate, those who reported that “I will definitely do this” and “I will probably do this” were coded as 1; those who reported “I will probably NOT do this” and “I will definitely NOT do this” were coded as 0. The early-intention-to-vaccinate measure had high internal consistency as assessed by Kuder–Richardson 20 (KR-20 = 0.79).

Covariates were included in the statistical analysis to minimize and adjust for potential confounding. The covariates included age, education, and income. Age was defined as the respondent’s age at the time of the survey. Based on median split, those aged 18–34 were coded as 1; those aged 35–64 were coded as 0. Education was defined as “last grade completed in school,” and the response was based on 9 categories: 8th grade or less; from 9th to 11th grade; high school graduate; GED; some college but no degree; associate degree—occupational/vocational; associate degree—academic program; bachelor’s degree; and graduate or professional school. Based on median split, those who reported having a bachelor’s degree or higher were coded as 1, and those who reported some college education or lower were coded as 0. Income was defined as the annual household income the individual reported at the time of the survey. Based on median split, those who reported earning USD 75,000 or more were coded as 1, and those who earned from USD 0 to USD 74,999 were coded as 0.

#### 2.3.2. Predictor Variables

##### Sexual Behavior

The survey assessed the respondents’ number of male sexual partners and condom use.

The number of sexual partners was defined as the number of male anal sex partners in the last 12 months. Those who reported having 3 or more sexual partners were coded as 1, and those with 2 or fewer sexual partners were coded as 0.

Condom use was defined as the frequency of condom use in the past 12 months, and the response was based on 4 categories: never, seldom, sometimes, and often. Using condoms never or seldom was coded as 1, and using condoms sometimes or often was coded as 0.

##### Health Behavior and Healthcare Access

For health behavior and healthcare access, we assessed whether respondents had a primary care provider that they saw in the past 12 months, had a pharmacy that they trusted to give them an HIV vaccine, had Medicaid health insurance, had employer health insurance, and used PrEP.

Respondents reporting visiting a primary care provider in the past 12 months were coded as 1, and those who did not were coded as 0.

Pharmacy trust was defined as whether the participant reported having a pharmacy that they trusted to give them a potential HIV vaccine shot and that they can visit. Respondents reporting that they had a trusted pharmacy were coded as 1, and those who did not were coded as 0.

Medicaid health insurance was defined as whether the participant reported having Medicaid health insurance. Respondents reporting that they had Medicaid were coded as 1, and those who did not were coded as 0.

Employer health insurance was defined as whether the participant reported having employer health insurance or not. Respondents reporting that they had employer health insurance were coded as 1, and those who did not were coded as 0.

Taking PrEP was defined as whether the respondent was taking Truvada or Descovy. Respondents reporting that they were taking PrEP were coded as 1, and those who did not were coded as 0.

##### Health Behavior Model Constructs

The survey assessed respondents’ perceived susceptibility to HIV, concealment of MSM identity, fear of HIV stigma, and perceived discrimination against African American MSM from receiving HIV prevention support.

Susceptibility to HIV was defined as how much the patient feels their actions make them susceptible to HIV [27]. The question was stated as follows: “Considering all of the different factors that may contribute to HIV, including your own past and present behavior, how likely would you be to get HIV?” Respondents reporting that they were very likely to get HIV or somewhat likely to get HIV were coded as 1, and those who felt that they were slightly likely to get HIV or not likely to get HIV at all were coded as 0.

Concealment of MSM identity was a 7-item scale that measured the extent to which respondents concealed their sexual orientation in their everyday lives. Responses were based on 4 categories: never, rarely, sometimes, and all the time (KR-20 = 0.87) [28]. Scores ranged from 7 to 28. Based on median split, scores ≥ 17 were categorized as indicating concealing MSM identity more and were coded as 1; scores < 17 indicated concealing MSM identity less and were coded as 0.

Concern about others knowing that they have had the HIV vaccine was based on a 6-item scale that measures the respondent’s concern about others finding out they received an HIV vaccine and was based on a negative, injunctive-norm scale (KR-20 = 0.84) [29]. Scores ranged from 6 to 24. The responses to the questions were based on 4 categories: disagree a lot, disagree a little, agree a little, and agree a lot. Based on median split, scores ≥ 3 indicated higher concern and were coded as 1; scores < 13 indicated lower concern and were coded as 0.

Fear of HIV stigma was based on a 6-item HIV stigma scale that assessed the seriousness of the negative consequences of having HIV (KR-20 = 0.85) [30]. Scores ranged from 6 to 24. The responses to the questions were based on 4 categories: disagree a lot, disagree a little, agree a little, and agree a lot. Based on median split, scores ≥ 18 indicated high fear of HIV stigma and were coded as 1, and scores < 18 indicated low fear of HIV stigma and were coded as 0.

Perceived discrimination against receiving HIV prevention support because of African American MSM identity was based on 4 items from the HIV stigma scale derived from the HIV-related perceived discrimination scale (KR-20 = 0.85) [27]. Scores ranged from 4 to 16. The responses to the questions were based on 4 categories: none, a little, some, and a lot. Based on median split, scores ≥ 11 indicated higher perceived discrimination and were coded as 1; scores < 11 indicated lower perceived discrimination and were coded as 0.

##### COVID-19 Vaccine History

We assessed the number of COVID-19 vaccinations received and the WHO Vaccine Positive Attitude Scale scores.

Respondents reporting 2 or more COVID-19 vaccinations were coded as 1, and those who received ≤ 1 COVID-19 shots were coded as 0.

The WHO HIV Vaccine Positive Attitude Scale is a 9-item scale derived from the WHO Vaccine Hesitancy Scale. The WHO Vaccine Hesitancy Scale was designed to measure parents’ hesitancy to have their children vaccinated [31]. We chose the scale because it was adapted into a general vaccine hesitancy scale to assess adults’ general attitudes about vaccines, and the scale was identified as valid across multiple studies [32]. In the present study, we observed high internal consistency between the scale items (KR-20 = 0.86). This study adapted the scale to specifically mention an HIV vaccine. We used the 7 positively worded questions about positive attitudes towards the vaccine to assess positive vaccine attitudes. Scores ranged from 7 to 49. The responses to the questions were based on 4 categories: disagree a lot, disagree a little, agree a little, and agree a lot. Based on median split, scores ≥ 23 indicated a more positive vaccine perception and were coded as 1; scores < 23 indicated a less positive vaccine perception and were coded as 0.

##### Mistrust

Medical mistrust was based on a 6-item scale that assessed perceptions of suspicion of the healthcare system by African Americans (KR-20 = 0.89) [33]. Scores ranged from 4 to 24. The responses to the questions were based on 4 categories: disagree a lot, disagree a little, agree a little, and agree a lot. Based on median split, scores ≥ 13 indicated high mistrust and were coded as 1; scores < 13 indicated low mistrust and were coded as 0.

Additionally, the survey assessed MSM group-based medical mistrust. This construct was measured with a 5-item scale that assessed parallel perceptions of suspicion of the healthcare system by African American MSM (KR-20 = 0.90) [33]. Scores ranged from 4 to 20. The responses to the questions were based on 4 categories: disagree a lot, disagree a little, agree a little, and agree a lot. Based on median split, scores ≥ 11 indicated high mistrust and were coded as 1; scores < 11 indicated low mistrust and were coded as 0.

### 2.4. Analysis

The study dichotomized each variable based on the classification described above. Contingency table analyses assessed the outcome variable, early intentions to vaccinate within one year, for associations with the predictors of interest. Associations identified as significant at *p* < 0.05 were fitted into a logistic regression model. The adjusted logistic model controlled for respondents’ age, education, and income. Model statistics included the adjusted odds ratio, 95% confidence interval, and corresponding *p*-value. We used STATA 17 to compute all statistical analyses.

## 3. Results

Table 1 displays descriptive statistics for sociodemographic and other characteristics by the intention to vaccinate. The majority (62%) of participants reported the desire to receive the HIV vaccine within one year if it was 90% effective against HIV, easy to obtain, free, and had few side effects. Most respondents were between 18 and 34 years of age, most had a Bachelor’s degree or higher, and a majority had household incomes of less than USD 75,000. Most participants (75%) had three or more male anal sex partners in the past 12 months, and most (76%) indicated that they sometimes or often used condoms. Most respondents (86%) had a primary care provider that they had seen in the last 12 months and a pharmacy that they trusted to provide the vaccine (82%). Nearly half (49%) of the respondents reported that they used PrEP, received Medicaid (45%), or had employer-provided health insurance (45%). The vast majority (84%) had received two or more COVID-19 vaccinations. In terms of HIV perception, some (31%) of the respondents felt that they were somewhat or very likely to get HIV, and some (45%) had significant concerns about others knowing that they had the HIV vaccine. Most (56%) had a high fear of HIV stigma. Most (54%) of the respondents felt the need to conceal their MSM identity, and most (53%) also felt that HIV prevention support discriminates against African American MSM individuals. Most respondents (59%) had positive HIV vaccine perceptions based on the WHO HIV Vaccine Positive Attitude Scale. In terms of medical mistrust, most felt high African American medical mistrust (56%) and MSM medical mistrust (52%).

The adjusted logistic regression model assessed the associations between the constructs and vaccine intentions (Table 2). Relative to African American MSM who reported that they would delay vaccination for more than one year, and controlling for age, education, and income, those who reported early vaccine intentions who had two or more COVID-19 vaccinations and those who reported early vaccine intentions who had high WHO HIV Vaccine Positive Attitude Scale scores were, respectively, 3.2 times and 2.4 times more likely to report early intentions to vaccinate within one year. Respondents who said that HIV prevention support discriminated against African American MSM individuals were also 2.4 times more likely to report early intentions to vaccinate within one year. Those who had three or more sexual partners and those reporting African American group-based medical mistrust were, respectively, 60% and 59% more likely to report delayed HIV vaccination intentions.

## 4. Discussion

This study identified a range of factors associated with the early intention to vaccinate against HIV. Several of these factors are modifiable. Two of the most robust predictors of the HIV vaccine intention were the vaccine attitude and prior vaccine history. Those receiving two or more COVID-19 vaccinations and those who had high WHO HIV Vaccine Positive Attitude Scale scores were over three and two times more likely, respectively, to have earlier HIV vaccination intentions. Those who felt that HIV prevention services discriminate against supporting the African American MSM community had nearly two and a half times greater early vaccine intentions. Two factors associated with delayed vaccine intentions were having three or more sexual partners and those who felt that African Americans should not trust the medical system. In the United States, the likelihood of African American MSM contracting HIV in their lifetimes is one in two, compared to a one in five risk among Latinx MSM and a one in eleven chance among white MSM [34]. Having HIV vaccine acceptance and uptake is crucial to addressing the disparate rates of HIV among the African American MSM community.

Health stakeholders can utilize those with predictor factors that influence high vaccine intentions for the initial HIV vaccination when the vaccine is released. Those seeking immediate HIV vaccination may be those frustrated by the lack and poor quality of HIV prevention services provided to the African American MSM community. African American MSM often face intense stigma when interacting with the United States healthcare system [13]. Racial biases often influence clinical decisions, acting as systemic obstacles to preventative measures, as evidenced by healthcare providers’ low willingness to prescribe PrEP to African American patients [35]. The majority of the respondents were young and they may lack resources (e.g., insurance, finances, transportation) to refer to HIV prevention services consistently or find that the support sites do not align with both their African American and MSM identities [36]. African American MSM individuals who felt discrimination were more likely to have earlier HIV vaccination intentions. This may be because they felt that HIV prevention services, HIV medical breakthroughs, HIV policies, and HIV research funding were not reaching their community. As only 51% of youth in the U.S. are aware of their HIV status, these individuals may feel that the HIV vaccine may provide protection against HIV and may be easier to obtain relative to other HIV prevention services [37]. Because current HIV prevention methods like PrEP require continuous use to remain effective, it may be more feasible to self-advocate for a vaccine instead of constantly struggling to receive HIV prevention support.

Vaccine perceptions and vaccine histories are strong indicators of HIV vaccine intentions. Studies have shown that COVID-19 uptake could be predicted by an individual’s flu vaccine history [38,39]. Hesitancy is broader than skepticism or refusal, as it is, rather, a continuum between unquestioningly accepting and completely refusing all vaccines [40,41]. Vaccine hesitancy and acceptance are influenced by an individual’s vaccine perception and the broader social processes that underpin the consequences and benefits of vaccine uptake compared to vaccine resistance [42]. Consequently, health stakeholders can target vaccine hesitancy to encourage vaccine uptake. Prior vaccine histories, such as having two or more COVID-19 vaccinations, may be a useful measure to assess the receptivity to other vaccines. While HIV is an infectious disease and not a communicable disease, a history of taking the COVID-19 vaccine was a strong predictor of early HIV vaccine intentions. Similarly, the WHO Vaccine Positive Attitude Scale was a strong predictor for HIV vaccine intentions, as it has demonstrated similar utility for other vaccines [32]. Further studies should seek to understand that the extant lessons learned from one type of vaccine research may be transferrable to other vaccines. If vaccine sentiments are transferrable, health stakeholders can potentially use prior vaccine histories and other vaccine communication and education strategies to improve HIV vaccine messaging and enhance vaccine uptake [43,44].

When providing the HIV vaccine, health stakeholders need to address the perceived barriers, such as feelings of discrimination from and a mistrust of health institutions. The mistrust of medical systems is attributable, in part, to historical events in the African American community and may increase cautiousness towards receiving an HIV vaccine [45]. In general, African Americans have higher rates of vaccine hesitancy compared to whites [46,47,48]. Studies show that African American group-based medical mistrust was associated with low COVID-19 vaccine uptake, and a similar pattern may potentially reduce HIV vaccine uptake [49]. Healthcare providers are key HIV prevention resources but often lack the ability to engage African American MSM in constructive HIV dialogue, creating a missed opportunity [50]. Because African Americans who feel less cared for by their physicians feel lower medical trust, creating a positive patient–provider environment is crucial to enhance vaccine intention and uptake [51].

When providing HIV prevention support, health providers need to be aware of how the intersecting, stigmatized identities of being African American and MSM may affect their openness to discussing HIV prevention strategies [52,53]. Providers should create an environment and language tone where their patients do not feel negatively judged for their sexual behaviors in addition to their race/ethnicity so that patients do not feel vulnerable to mistreatment, stigma, and discrimination [54]. Investing in community dialogue regarding vaccine perceptions and concerns may further support providers in tailoring their HIV vaccine communication [55]. Encouraging African American MSM trust in health institutions providing the HIV vaccine may be crucial to enhancing the HIV vaccine uptake.

The rate of African American MSM participation in HIV vaccine clinical trials does not reflect their burden of HIV infection, and few interventions document the use of extensive strategies to recruit and retain racial and ethnic minorities [56,57]. The lack of recruitment and retention of African American MSM in HIV vaccine clinical trials may further exacerbate the feelings that the health and policy system discriminate against supporting African American MSM accessibility to HIV medical breakthroughs, as reported by African American MSM in this study. The findings of this study are also applicable to predicting factors that would assist clinical trial teams in recruiting and retaining African American MSM participants. Working with the community to develop the HIV vaccine is crucial to dispelling mistrust and creating vaccine support tailored to the community’s needs [58,59]. Health stakeholders should be aware of and have the capacity to address concerns that the HIV vaccines may create an “antibody-induced seropositivity”, which could increase discrimination against this population [60].

One counterintuitive finding was the relationship between the number of sex partners and HIV vaccine intentions. African American MSM who had three or more sexual partners were 60% less likely to receive early vaccination. This finding warrants further investigation. The low awareness and prioritization of the perceived HIV risks expressed by African American MSM highlight the need for more salient and tailored messaging and prevention support [61]. It is important to note that the findings and messaging should not solely target African American MSM, as doing so may further encourage the negative perception that these vaccine predictors are only relevant to this minoritized community. When creating African American MSM PrEP messaging, many African American MSM have stated that they may be more inclined to access PrEP if the messaging did not only feature African American MSM, as it worsens the perceived and experienced stigma that the community already faces [61].

### Limitations and Future Directions

This study has several methodological limitations. The data are from a convenience sample. As the respondents were mostly in their youth/early adulthood, the findings cannot be generalized to older age groups. However, we believe that it is important to study young adults, as this is a critical age at which to promote positive health behaviors to reduce potential lifelong adverse health outcomes. Additionally, this study is also bound by race and gender. The study chose to focus solely on African American MSM, as this is the population that is at the greatest risk for HIV infection in the U.S.; furthermore, they are often overlooked in research or are compared to “white” as the norm, which may inadvertently reinforce negative stereotypes [57]. Even studies that include African Americans often have small samples of African American MSM, limiting meaningful statistical analysis and racial/ethnic comparisons. The study is also geographically limited, as the respondents were required to reside in the United States at the time of the survey. Future studies should include greater specificity in geographic location to understand vaccine intention variations between urban and rural areas. The sample was recruited among those using computers or smart phones; hence, the respondents may be of a higher SES than those recruited from non-digital platforms. People who go online may also have higher levels of education and income.

The findings are potentially limited by recall bias, as the interview data were solely derived from self-reporting. To mitigate the effect of response bias, the study included various scales to measure the response consistency. To limit nonresponses, the survey was administered via a confidential survey on Qualtrics to enhance feelings of confidentiality and promote more candid responses to sensitive questions. Lastly, given the cross-sectional nature of the analysis, we cannot establish temporal ordering between the predictors and the outcomes, nor do we have measures of the extent to which vaccine intentions lead to actual vaccine behaviors. The HIV vaccine described in this study is hypothetical and was defined as highly effective with a low risk for adverse side effects, so the respondents may not respond similarly if an actual vaccine, with a different effectiveness and safety profile, is released.

## 5. Conclusions

There are several modifiable factors associated with the uptake of a hypothetical HIV vaccine among African American MSM. The lack of a knowledge base on HIV vaccine perception and acceptability is a missed opportunity to help health stakeholders, such as health providers and policymakers, develop and implement community and media programs to overcome HIV vaccine hesitancy once a vaccine is licensed. Developing diverse strategies to enhance these factors would be valuable in promoting the HIV vaccine uptake among this vulnerable population.

## Figures and Tables

**Table 1:** Bivariate factors associated with vaccine uptake.

	Total	Vaccination within One Year			
		No	Yes	Chi-2	
Variables	*N* (%)	*n* (%)	*n* (%)		*p*-value
Total	432 (100%)	166 (38.4%)	266 (61.6%)		
Age				0.045	0.833
35–64	96 (22.2%)	36 (37.5%)	60 (62.5%)		
18–34	336 (77.8%)	130 (38.7%)	206 (61.3%)		
Education				19.188	0.0001
Some college education or lower	175 (40.8%)	89 (50.9%)	86 (49.1%)		
Bachelor’s degree or higher	254 (59.2%)	76 (29.9%)	178 (70.1%)		
Income				18.699	0.0001
USD 0–74,999	229 (53.1%)	110 (48.0%)	119 (52.0%)		
USD 75,000 or more	202 (46.9%)	56 (27.7%)	146 (72.3%)		
Primary Care Provider In Past 12 Months				0.346	0.557
Did not see primary care provider	60 (13.9%)	21 (35.0%)	39 (65.0%)		
Did see primary care provider	372 (86.1%)	145 (39.0%)	227 (61.0%)		
Pharmacy Trust				0.973	0.324
No trusted pharmacy or not sure	76 (17.6%)	33 (43.4%)	43 (56.6%)		
Have a trusted pharmacy	356 (82.4%)	133 (37.4%)	223 (62.6%)		
Medicaid Health Insurance				0.472	0.492
Do not receive Medicaid	238 (55.1%)	88 (37.0%)	150 (63.0%)		
Receive Medicaid	194 (44.9%)	78 (40.2%)	116 (59.8%)		
Employer Health Insurance				0.583	0.445
No insurance through employer	239 (55.3%)	88 (36.8%)	151 (63.2%)		
Insurance through employer	193 (44.7%)	78 (40.4%)	115 (59.6%)		
Condom Use				0.954	0.329
Often or sometimes use condoms	330 (76.4%)	131 (39.7%)	199 (60.3%)		
Never or rarely use condoms	102 (23.6%)	35 (34.3%)	67 (65.7%)		
Perceived Susceptibility to HIV				0.376	0.54
Feel will not or are slightly likely to get HIV	297 (68.8%)	117 (39.4%)	180 (60.6%)		
Feel somewhat or very likely to get HIV	135 (31.3%)	49 (36.3%)	86 (63.7%)		
Concealment of MSM Identity				6.889	0.009
Conceal MSM identity less	201 (46.5%)	64 (31.8%)	137 (68.2%)		
Conceal MSM identity more	231 (53.5%)	102 (44.2%)	129 (55.8%)		
Concern About Others Knowing They Had HIV Vaccine				24.555	0.0001
Low concern	237 (54.9%)	50 (25.6%)	145 (74.4%)		
High concern	195 (45.1%)	116 (49.0%)	121 (51.1%)		
Fear of HIV Stigma				0.024	0.877
Low fear	192 (44.4%)	73 (38.0%)	119 (62.0%)		
High fear	240 (55.6%)	93 (38.8%)	147 (61.3%)		
Feel Discrimination Against African American MSM for HIV Support				24.969	0.0001
Feel low discrimination	205 (47.5%)	104 (50.7%)	101 (49.3%)		
Feel high discrimination	227 (52.6%)	62 (27.3%)	165 (72.7%)		
Multiple Male Partners				5.373	0.02
Two partners	107 (24.8%)	31 (29.0%)	76 (71.0%)		
Three or more partners	325 (75.2%)	135 (41.5%)	190 (58.5%)		
Take PrEP				1.270	0.26
No	222 (51.4%)	91 (41.0%)	131 (59.0%)		
Yes	210 (48.6%)	75 (35.7%)	135 (64.3%)		
Had Two or More COVID-19 Shots				17.482	0.0001
No	69 (16.0%)	42 (60.9%)	27 (39.1%)		
Yes	363 (84.0%)	124 (34.2%)	239 (65.8%)		
WHO HIV Vaccine Positive Attitude Scale				41.388	0.0001
Poor HIV vaccine perception	177 (41.0%)	100 (56.5%)	77 (43.5%)		
Positive HIV vaccine perception	255 (59.0%)	66 (25.9%)	189 (74.1%)		
Group-Based Medical Mistrust (as Black)				16.912	0.0001
Low mistrust	189 (43.8%)	52 (27.5%)	137 (72.5%)		
High mistrust	243 (56.3%)	114 (46.9%)	129 (53.1%)		
Group-Based Medical Mistrust (as MSM)				4.678	0.031
Low mistrust	208 (48.2%)	69 (33.2%)	139 (66.8%)		
High mistrust	224 (51.9%)	97 (43.3%)	127 (56.7%)		

**Table 2:** Multiple logistic regression describing the association of constructs with HIV vaccination intentions.

		Vaccination within One Year		
Variables	Odds Ratio	95% Conf. Interval		*p*-Value
Age: 18–34 ^1^	1.28	0.73	2.24	0.389
Income: USD 75,000 or more ^2^	1.68	0.99	2.86	0.055
Education: bachelor’s degree or higher ^3^	1.20	0.68	2.1	0.532
Concern about others knowing they received the HIV vaccine ^4^	1.64	0.99	2.74	0.057
Concealment of MSM identity ^5^	0.62	0.38	1.02	0.062
Feels high discrimination against receiving HIV prevention support because of African American MSM identity ^6^	2.43	1.49	3.97	0.0001
Three or more sexual partners ^7^	0.40	0.23	0.69	0.001
Two or more COVID-19 shots ^8^	3.19	1.71	5.96	0.0001
High WHO HIV Vaccine Positive Attitude Scale score ^9^	2.40	1.42	4.06	0.001
High group-based medical distrust (as African American) ^10^	0.41	0.21	0.8	0.009
High group-based medical distrust (as MSM) ^11^	1.22	0.60	2.47	0.576

Reference levels for key variables: ^1^ age: 35–64; ^2^ income: USD 0–74,999; ^3^ some college education or lower; ^4^ low concern about others knowing they have HIV; ^5^ conceals MSM identity less; ^6^ feels low discrimination against receiving HIV prevention support because of African American MSM identity; ^7^ two or less sexual partners; ^8^ less than two COVID-19 shots; ^9^ low WHO HIV Vaccine Positive Attitude Scale score; ^10^ low group-based medical distrust (as African American); ^11^ low group-based medical distrust (as MSM).

## Data Availability

Please contact the author for the data.

## References

[1] WHO. HIV. The Global Health Observatory. 2023. https://www.who.int/data/gho/data/themes/hiv-aids.

[2] Bekker L.-G., Beyrer C., Mgodi N., Lewin S.R., Delany-Moretlwe S., Taiwo B., Masters M.C., Lazarus J.V. (2023). HIV infection. Nat. Rev. Dis. Primers.

[3] Landovitz R.J., Scott H., Deeks S.G. (2023). Prevention, treatment and cure of HIV infection. Nat. Rev. Microbiol..

[4] Jain S., Venkataraman A., Wechsler M.E., Peppas N.A. (2021). Messenger RNA-based vaccines: Past, present, and future directions in the context of the COVID-19 pandemic. Adv. Drug Deliv. Rev..

[5] Kay E.S., Pinto R.M. (2020). Is Insurance a Barrier to HIV Preexposure Prophylaxis? Clarifying the Issue. Am. J. Public Health.

[6] Fauci A.S., Lane H.C. (2020). Four Decades of HIV/AIDS—Much Accomplished, Much to Do. N. Engl. J. Med..

[7] Houser K.V., Gaudinski M.R., Happe M., Narpala S., Verardi R., Sarfo E.K., Corrigan A.R., Wu R., Rothwell R.S., Novik L. (2022). Safety and immunogenicity of an HIV-1 prefusion-stabilized envelope trimer (Trimer 4571) vaccine in healthy adults: A first-in-human open-label, randomized, dose-escalation, phase 1 clinical trial. eClinicalMedicine.

[8] Matarazzo L., Bettencourt P.J.G. (2023). mRNA vaccines: A new opportunity for malaria, tuberculosis and HIV. Front. Immunol..

[9] Excler J.L., Saville M., Privor-Dumm L., Gilbert S., Hotez P.J., Thompson D., Abdool-Karim S., Kim J.H. (2023). Factors, enablers and challenges for COVID-19 vaccine development. BMJ Glob Health.

[10] Fortner A., Bucur O. (2022). mRNA-based vaccine technology for HIV. Discoveries.

[11] CDC HIV among Gay and Bisexual Men in the U.S. CHHSTP Newsroom. 2022. https://www.cdc.gov/nchhstp/newsroom/fact-sheets/hiv/HIV-gay-bisexual-men.html#print.

[12] Frye V., Nandi V., Egan J., Cerda M., Greene E., Van Tieu H., Ompad D.C., Hoover D.R., Lucy D., Baez E. (2015). Sexual Orientation- and Race-Based Discrimination and Sexual HIV Risk Behavior Among Urban MSM. AIDS Behav..

[13] Mayer K.H., Nelson L., Hightow-Weidman L., Mimiaga M.J., Mena L., Reisner S., Daskalakis D., Safren S.A., Beyrer C., Sullivan P.S. (2021). The persistent and evolving HIV epidemic in American men who have sex with men. Lancet.

[14] Matthews D.D., Herrick A.L., Coulter R.W., Friedman M.R., Mills T.C., Eaton L.A., Wilson P.A., Stall R.D. (2016). Running Backwards: Consequences of Current HIV Incidence Rates for the Next Generation of Black MSM in the United States. AIDS Behav..

[15] Levy M.E., Wilton L., Phillips G., Glick S.N., Kuo I., Brewer R.A., Elliott A., Watson C., Magnus M. (2014). Understanding Structural Barriers to Accessing HIV Testing and Prevention Services Among Black Men Who Have Sex with Men (BMSM) in the United States. AIDS Behav..

[16] Mohamed K., Rzymski P., Islam M.S., Makuku R., Mushtaq A., Khan A., Ivanovska M., Makka S.A., Hashem F., Marquez L. (2022). COVID-19 vaccinations: The unknowns, challenges, and hopes. J. Med. Virol..

[17] Sallam M. (2021). COVID-19 Vaccine Hesitancy Worldwide: A Concise Systematic Review of Vaccine Acceptance Rates. Vaccines.

[18] Hickler B., Guirguis S., Obregon R. (2015). Vaccine Special Issue on Vaccine Hesitancy. Vaccine.

[19] Torracinta L., Tanner R., Vanderslott S. (2021). MMR Vaccine Attitude and Uptake Research in the United Kingdom: A Critical Review. Vaccines.

[20] Mereu N., Mereu A., Murgia A., Liori A., Piga M., Argiolas F., Salis G., Santus S., Porcu C., Contu P. (2020). Vaccination Attitude and Communication in Early Settings: An Exploratory Study. Vaccines.

[21] Maiman L.A., Becker M.H. (1974). The Health Belief Model: Origins and Correlates in Psychological Theory. Health Educ. Monogr..

[22] Sheeran P., Abraham C. (1996). The health belief model. Predict. Health Behav..

[23] Janz N.K., Becker M.H. (1984). The Health Belief Model: A Decade Later. Health Educ. Q..

[24] Maloney E.K., Lapinski M.K., Witte K. (2011). Fear Appeals and Persuasion: A Review and Update of the Extended Parallel Process Model. Soc. Personal. Psychol. Compass.

[25] Nomura S., Eguchi A., Yoneoka D., Kawashima T., Tanoue Y., Murakami M., Sakamoto H., Maruyama-Sakurai K., Gilmour S., Shi S. (2021). Reasons for being unsure or unwilling regarding intention to take COVID-19 vaccine among Japanese people: A large cross-sectional national survey. Lancet Reg. Health—West. Pac..

[26] Hair J.F., Babin B.J., Black W.C. (2019). Multivariate Data Analysis.

[27] Hoyt M.A., Rubin L.R., Nemeroff C.J., Lee J., Huebner D.M., Proeschold-Bell R.J. (2012). HIV/AIDS-related institutional mistrust among multiethnic men who have sex with men: Effects on HIV testing and risk behaviors. Health Psychol..

[28] Jackson S.D., Mohr J.J. (2016). Conceptualizing the closet: Differentiating stigma concealment and nondisclosure processes. Psychol. Sex. Orientat. Gend. Divers..

[29] Lee S.J., Newman P.A., Duan N., Cunningham W.E. (2014). Development of an HIV vaccine attitudes scale to predict HIV vaccine acceptability among vulnerable populations: L.A. VOICES. Vaccine.

[30] Golub S.A., Gamarel K.E. (2013). The Impact of Anticipated HIV Stigma on Delays in HIV Testing Behaviors: Findings from a Community-Based Sample of Men Who Have Sex with Men and Transgender Women in New York City. AIDS Patient Care STDs.

[31] Larson H.J., Jarrett C., Schulz W.S., Chaudhuri M., Zhou Y., Dube E., Schuster M., MacDonald N.E., Wilson R. (2015). Measuring vaccine hesitancy: The development of a survey tool. Vaccine.

[32] Luyten J., Bruyneel L., van Hoek A.J. (2019). Assessing vaccine hesitancy in the UK population using a generalized vaccine hesitancy survey instrument. Vaccine.

[33] Thompson H.S., Valdimarsdottir H.B., Winkel G., Jandorf L., Redd W. (2004). The Group-Based Medical Mistrust Scale: Psychometric properties and association with breast cancer screening. Prev. Med..

[34] Hess K.L., Hu X., Lansky A., Mermin J., Hall H.I. (2017). Lifetime risk of a diagnosis of HIV infection in the United States. Ann. Epidemiol..

[35] Calabrese S.K., Earnshaw V.A., Underhill K., Hansen N.B., Dovidio J.F. (2014). The Impact of Patient Race on Clinical Decisions Related to Prescribing HIV Pre-Exposure Prophylaxis (PrEP): Assumptions About Sexual Risk Compensation and Implications for Access. AIDS Behav..

[36] Beach L.B., Greene G.J., Lindeman P., Johnson A.K., Adames C.N., Thomann M., Washington P.C., Phillips G. (2018). Barriers and Facilitators to Seeking HIV Services in Chicago Among Young Men Who Have Sex with Men: Perspectives of HIV Service Providers. AIDS Patient Care STDs.

[37] Lally M.A., van den Berg J.J., Westfall A.O., Rudy B.J., Hosek S.G., Fortenberry J.D., Monte D., Tanney M.R., McFarland E.J., Xu J. (2018). HIV Continuum of Care for Youth in the United States. JAIDS J. Acquir. Immune Defic. Syndr..

[38] Willis D.E., Selig J.P., Andersen J.A., Hall S., Hallgren E., Williams M., Bryant-Moore K., McElfish P.A. (2023). Hesitant but vaccinated: Assessing COVID-19 vaccine hesitancy among the recently vaccinated. J. Behav. Med..

[39] Campbell J., Kaur A., Gamino D., Benoit E., Amos B., Windsor L. (2023). Individual and structural determinants of COVID-19 vaccine uptake in a marginalized community in the United States. Vaccine.

[40] MacDonald N.E. (2015). Vaccine hesitancy: Definition, scope and determinants. Vaccine.

[41] Martin L.R., Petrie K.J. (2017). Understanding the Dimensions of Anti-Vaccination Attitudes: The Vaccination Attitudes Examination (VAX) Scale. Ann. Behav. Med..

[42] Brewer N.T., Chapman G.B., Rothman A.J., Leask J., Kempe A. (2017). Increasing Vaccination: Putting Psychological Science Into Action. Psychol. Sci. Public Interest.

[43] Shen A.K., Browne S., Srivastava T., Kornides M.L., Tan A.S.L. (2023). Trusted messengers and trusted messages: The role for community-based organizations in promoting COVID-19 and routine immunizations. Vaccine.

[44] Xu M.A., Choi J., Capasso A., DiClemente R. (2023). Patient-Provider Health Communication Strategies: Enhancing HPV Vaccine Uptake among Adolescents of Color. Healthcare.

[45] Willis D.E., Andersen J.A., Bryant-Moore K., Selig J.P., Long C.R., Felix H.C., Curran G.M., McElfish P.A. (2021). COVID-19 vaccine hesitancy: Race/ethnicity, trust, and fear. Clin. Transl. Sci..

[46] Abba-Aji M., Stuckler D., Galea S., McKee M. (2022). Ethnic/racial minorities’ and migrants’ access to COVID-19 vaccines: A systematic review of barriers and facilitators. J. Migr. Health.

[47] Jamison A.M., Quinn S.C., Freimuth V.S. (2019). “You don’t trust a government vaccine”: Narratives of institutional trust and influenza vaccination among African American and white adults. Soc. Sci. Med..

[48] Majee W., Anakwe A., Onyeaka K., Harvey I.S. (2023). The Past Is so Present: Understanding COVID-19 Vaccine Hesitancy Among African American Adults Using Qualitative Data. J. Racial Ethn. Health Disparities.

[49] Morgan K.M., Maglalang D.D., Monnig M.A., Ahluwalia J.S., Avila J.C., Sokolovsky A.W. (2023). Medical Mistrust, Perceived Discrimination, and Race: A Longitudinal Analysis of Predictors of COVID-19 Vaccine Hesitancy in US Adults. J. Racial Ethn. Health Disparities.

[50] Fields E.L., Hussen S.A., Malebranche D.J. (2020). Mind the Gap: HIV Prevention Among Young Black Men Who Have Sex with Men. Curr. HIV/AIDS Rep..

[51] Martin K.J., Stanton A.L., Johnson K.L. (2023). Current health care experiences, medical trust, and COVID-19 vaccination intention and uptake in Black and White Americans. Health Psychol..

[52] Elopre L., Hussen S.A., Ott C., Mugavero M.J., Turan J.M. (2021). A Qualitative Study: The Journey to Self-Acceptance of Sexual Identity among Young, Black MSM in the South. Behav. Med..

[53] Babel R.A., Wang P., Alessi E.J., Raymond H.F., Wei C. (2021). Stigma, HIV Risk, and Access to HIV Prevention and Treatment Services Among Men Who have Sex with Men (MSM) in the United States: A Scoping Review. AIDS Behav..

[54] Threats M., Boyd D.T., Diaz J.E., Adebayo O.W. (2021). Deterrents and motivators of HIV testing among young Black men who have sex with men in North Carolina. AIDS Care.

[55] Schoch-Spana M., Brunson E.K., Long R., Ruth A., Ravi S.J., Trotochaud M., Borio L., Brewer J., Buccina J., Connell N. (2021). The public’s role in COVID-19 vaccination: Human-centered recommendations to enhance pandemic vaccine awareness, access, and acceptance in the United States. Vaccine.

[56] Bass S.B., D’Avanzo P., Alhajji M., Ventriglia N., Trainor A., Maurer L., Eisenberg R., Martinez O. (2020). Exploring the Engagement of Racial and Ethnic Minorities in HIV Treatment and Vaccine Clinical Trials: A Scoping Review of Literature and Implications for Future Research. AIDS Patient Care STDs.

[57] Watson C.C., Wilton L., Lucas J.P., Bryant L., Victorianne G.D., Aradhya K., Fields S.D., Wheeler D.P., on behalf of the HPTN Black Caucus (2020). Development of a Black Caucus within the HIV Prevention Trials Network (HPTN): Representing the Perspectives of Black Men Who Have Sex with Men (MSM). Int. J. Environ. Res. Public Health.

[58] Below J.E., Fisher-Hoch S.P., McCormick J.B., North K.E. (2023). Challenges and strategies for recruitment of minorities to clinical research and trials. J. Clin. Transl. Sci..

[59] Tuckerman J., Kaufman J., Danchin M. (2022). Effective Approaches to Combat Vaccine Hesitancy. Pediatr. Infect. Dis. J..

[60] Dhalla S., Poole G. (2011). Barriers of enrolment in HIV vaccine trials: A review of HIV vaccine preparedness studies. Vaccine.

[61] Elopre L., Ott C., Lambert C.C., Amico K.R., Sullivan P.S., Marrazzo J., Mugavero M.J., Turan J.M. (2021). Missed Prevention Opportunities: Why Young, Black MSM with Recent HIV Diagnosis did not Access HIV Pre-exposure Prophylaxis Services. AIDS Behav..

